# Comparison of the concomitant use of anti-obesity medications with endoscopic sleeve gastroplasty versus endoscopic sleeve gastroplasty alone: a systematic review and meta-analysis

**DOI:** 10.1007/s00464-026-12936-w

**Published:** 2026-07-24

**Authors:** Luiz Gustavo de Quadros, Manoel Galvão Neto, Caio Colturato Coimbra, André Teixieira, Admar Concon Filho, Roberto Luiz Kaiser Junior, Martins Fideles dos Santos Neto, Carolina Colombelli Pacca, Eduardo Grecco

**Affiliations:** 1 Faculdade de Medicina de São José do Rio Preto, Programa de Pós-graduação em Ciências da Saúde, São José do Rio Preto, Brazil; 2 Kaiser Hospital, São José do Rio Preto, Brazil; 3 Faculdade de Medicina do ABC, Departamento de Endoscopia Digestiva, Santo André, São Paulo, Brazil; 4 Orlando Health, Orlando, FL USA; 5 Clínica Concon, Valinhos, SP Brazil; 6 Research Group GEISATEC—Health Innovation, Technology and Management—Barretos Cancer Hospital, Rio de Janeiro, Brazil; 7 Unesp, Programa de Pós-Graduação em Microbiologia, São José do Rio Preto, SP Brazil

**Keywords:** Endoscopic sleeve gastroplasty (ESG), Anti-obesity medications (AOMs), Combination therapy, Weight loss and metabolic outcomes

## Abstract

**Background:**

Endoscopic sleeve gastroplasty (ESG) is increasingly combined with anti-obesity medications (AOMs), but the incremental benefit of combination therapy over ESG alone remains uncertain

**Methods:**

This systematic review and meta-analysis followed PRISMA guidelines and was registered in PROSPERO (CRD420251179747). Comparative studies evaluating ESG plus AOMs versus ESG alone in adults with overweight or obesity were included. Random-effects models were used for pooled analyses

**Results:**

Ten studies comprising 578 participants met inclusion criteria. At approximately seven months, combination therapy showed a modest, non-significant advantage in total weight loss (mean difference +1.9% TWL; 95% CI − 1.22 to 4.99), with substantial heterogeneity (I^2^ ≈ 80–90%). For metabolic outcomes, the pooled mean difference in HbA1c change was minimal (− 0.04%; 95% CI − 0.37 to 0.28; *p* = 0.79), and individual studies reported inconsistent effects

**Conclusions:**

ESG combined with AOMs may enhance early weight loss in selected settings; however, current evidence does not support consistent long-term superiority over ESG alone for weight or glycemic outcomes. Although the available data remain limited and heterogeneous, concomitant therapy may be considered in selected patients rather than as a universally superior strategy. Multicenter randomized trials with standardized pharmacotherapy protocols, longer follow-up, and improved patient stratification are needed to clarify durability, cost-effectiveness, and the subgroups most likely to benefit.

Obesity has emerged as one of the most pressing global health challenges of the 21st century, characterized by its complex, multifactorial, and chronic nature [1–4]. Despite advances in preventive and therapeutic strategies, its prevalence continues to rise worldwide, contributing to increased rates of metabolic disorders, cardiovascular diseases, and healthcare expenditures [5, 6]. In parallel with the growing recognition of obesity as a chronic disease requiring long-term and multimodal management, minimally invasive and pharmacological approaches have gained increasing prominence within contemporary treatment algorithms. In this context, endoscopic sleeve gastroplasty (ESG) has become an important bariatric endoscopic option because it promotes gastric remodeling, induces clinically meaningful weight loss, and has shown a favorable safety profile in appropriately selected patients [7–9]. Concurrently, anti-obesity medications (AOMs), particularly glucagon-like peptide-1 receptor agonists (GLP-1 RAs), have expanded the therapeutic landscape of obesity care by offering pharmacological support for appetite regulation, weight reduction, and metabolic improvement [10–12].

As both modalities have become more widely incorporated into clinical practice, their combined use has increasingly entered therapeutic reasoning, especially in scenarios where a multimodal strategy appears conceptually attractive or clinically desirable [13, 14]. This movement, however, raises an important practical and scientific issue. The adoption of ESG together with AOMs has progressed more rapidly than the consolidation of comparative evidence capable of demonstrating whether such concomitant use confers a consistent incremental benefit over ESG alone. Thus, the clinical appeal of combining an endoscopic restrictive intervention with adjunct pharmacotherapy has, to some extent, preceded the availability of robust and integrated evidence to support broad recommendations. This mismatch is particularly relevant in a field where treatment decisions involve not only expectations of greater weight loss, but also additional cost, therapeutic complexity, drug tolerability, adherence burden, and long-term care planning [15–17].

The relevance of this question is further amplified by the nature of the currently available literature. The gap in knowledge is not merely quantitative, in the sense of there being relatively few comparative studies, but also structural, because the emerging evidence remains dispersed, methodologically heterogeneous, and partially confined to gray literature, including conference abstracts. As a result, clinicians and investigators may encounter preliminary signals suggesting potential benefit from combination therapy, yet still lack a focused, critical, and methodologically organized synthesis capable of clarifying the direction, consistency, and practical meaning of those findings. In this setting, biologically plausible synergy should not be assumed to represent demonstrated clinical superiority. Although ESG and AOMs may theoretically complement one another through different and potentially convergent mechanisms related to gastric restriction, appetite regulation, and metabolic modulation [18, 19], physiopathological plausibility alone does not replace direct comparative evidence. A rigorous synthesis is therefore necessary not only to gather available studies, but also to critically examine whether an intuitively promising strategy is truly supported by the current literature.

From a clinical perspective, this question has immediate relevance, not because it permits a definitive practice-changing recommendation, but because it informs decision-making in an area where therapeutic incorporation is already occurring under conditions of uncertainty. In other words, the practical value of this topic lies less in determining that ESG plus AOMs should be routinely adopted for all patients and more in clarifying whether the available evidence is sufficient to justify such generalization. This distinction is important for patient selection, counseling, expectation management, treatment sequencing, and individualized long-term follow-up. It is also relevant to broader considerations in obesity care, including cost-effectiveness, escalation of treatment burden, and the identification of subgroups in whom adjunct pharmacotherapy may be more appropriately considered rather than universally prescribed [20, 21]. Accordingly, a study addressing this comparison may be clinically valuable not only for what it confirms, but also for what it delineates as unresolved, thereby helping to organize a decision that is already being made in real-world practice.

In addition, the present review has originality in its analytical framing. Its contribution does not lie in discussing ESG in isolation, nor in broadly revisiting combination therapies for obesity, but in specifically focusing on the direct comparative question of ESG combined with anti-obesity medication versus ESG alone. This is precisely the question that emerges in clinical consultation, therapeutic planning, and longitudinal obesity management, yet it has remained insufficiently resolved by the existing literature. By addressing this focused comparison, the present study seeks to transform fragmented and early-stage evidence into a coherent analytical framework, while also identifying the limits that currently prevent more definitive conclusions. Therefore, this systematic review and meta-analysis aimed to synthesize the available evidence comparing the efficacy of concomitant ESG and anti-obesity medication versus ESG alone, with particular attention to its implications for clinical interpretation, therapeutic individualization, and the future research agenda in this evolving field.

## Methods

### Protocol registration

This systematic review, developed in accordance with the preferred reporting items for systematic reviews and meta-analyses (PRISMA) guidelines, was prospectively registered in the international prospective register of systematic reviews (PROSPERO) under registration number CRD420251179747.

### Research question

This systematic review was structured according to the PICO framework to address the following research question: Among adults with overweight or obesity, does the concomitant use of anti-obesity pharmacotherapy with ESG result in greater weight loss and metabolic improvement compared to ESG alone? The Population comprises adult patients diagnosed with overweight or obesity, with or without metabolic comorbidities. The Intervention involves ESG performed in conjunction with pharmacological therapy using approved anti-obesity agents. The Comparison group includes patients undergoing ESG as a standalone procedure. The Outcomes of interest include the percentage of total weight loss (%TWL) and the variation in glycated hemoglobin (HbA1c %), which together reflect the effectiveness of the intervention in promoting weight reduction and improving glycemic control.

### Search strategy and databases

The search strategy was systematically developed using controlled vocabularies from both Medical Subject Headings (MeSH) and Emtree to ensure comprehensive retrieval of relevant studies. Boolean operators “OR” and “AND” were employed to appropriately combine terms and refine the search logic. The strategy was adapted to the indexing structure of each database to maximize sensitivity and specificity. A specialized health sciences librarian provided methodological guidance in constructing and validating the search strategy. Thus, the final strategy was: ("Endoscopic Gastroplasty" OR "Endoluminal Gastroplasty" OR "Endoscopic Sleeve Gastroplasty (ESG)" OR "Endoscopic Gastric Plication" OR "Endoscopic Gastric Sleeve" OR "Endoscopic Stomach Plication" OR "Endoscopic Stomach Reduction" OR "Endoscopic Gastric Remodeling" OR "Endoscopic Suturing Gastroplasty" OR "OverStitch Endoscopic Gastroplasty" OR "Full-Thickness Endoscopic Plication" OR "Transoral Endoscopic Gastroplasty" OR "Endoluminal Sleeve Gastroplasty" OR "Minimally Invasive Endoscopic Gastric Reduction" OR "Endoscopic Gastric Volume Reduction" OR "Endoscopic Bariatric Gastroplasty" OR "Endoscopic Gastric Tightening Procedure") AND (Semaglutide OR Liraglutide OR Dulaglutide OR Exenatide OR Lixisenatide OR Albiglutide OR Tirzepatide OR Retatrutide OR Cotadutide OR Danuglipron OR “Combined therapy” OR Medication).

The electronic databases consulted included PubMed, Scopus, Embase, Cochrane Library, Web of Science, Virtual Health Library (BVS), and LILACS. In addition, grey literature was explored through the CAPES Theses and Dissertations Catalog to minimize publication bias.

### Eligibility criteria

Eligible studies included original research involving adult human participants (≥18 years) who underwent endoscopic sleeve gastroplasty (ESG) either as a standalone intervention and in combination with anti-obesity pharmacotherapy, with outcomes related to total weight loss (%TWL) or glycemic control (HbA1c%). No restrictions were applied regarding publication year or language, although search descriptors were formulated in English. Studies were excluded if they: (1) involved animal models or in vitro experiments; (2) presented duplicate data; (3) described ESG and pharmacological treatment without evaluating their combined use; (4) focused primarily on technical aspects of ESG or on the pharmacodynamic effects of medications rather than comparative outcomes; or (5) addressed cost-effectiveness analyses only. While conference abstracts are typically excluded from systematic reviews, they were considered eligible in this study due to the limited number of publications examining the concomitant use of ESG and anti-obesity medications. Given the scarcity of directly comparative studies in this field, excluding conference abstracts would likely have resulted in an incomplete representation of the available evidence and would have further limited the ability of this review to characterize current research trends. Therefore, in this specific context, abstracts were considered eligible only when they provided sufficient comparative data for analysis. Although this approach departs from conventional systematic review practice and was interpreted with caution, it was adopted to ensure a broader and more informative synthesis of an emerging literature, while also helping to identify areas in which more robust full-text studies are urgently needed.

### Study selection

After the exclusion of duplicate records, the study selection process was carried out in two sequential phases: an initial screening of titles and abstracts, followed by a comprehensive full-text review of potentially eligible articles. Two independent reviewers (M.F.S.N. and A.S.) applied the predefined inclusion and exclusion criteria throughout this process. Any disagreements regarding study eligibility were addressed through consensus or resolved with the input of a third reviewer (P.R.J.D.). When different publications presented overlapping datasets, only the most recent version was retained to maintain analytical accuracy and avoid redundancy.

### Risk of bias

The methodological quality of the included studies was appraised using the Newcastle–Ottawa quality assessment scale (NOS), appropriate for evaluating cohort and case-control designs. This choice reflects the predominantly observational nature of bariatric endoscopic research, which relies on real-world longitudinal data to assess clinical effectiveness and safety across heterogeneous populations.

### Data extraction

Data extraction was conducted independently by two reviewers using EndNote X9 to manage article metadata, remove duplicates, and organize eligible studies. The process was complemented by Microsoft Excel, which was used for structured extraction and analysis of study variables. The variables were selected by field specialists according to the research objectives and included: Author/Year, Population (ESG + Medication), Type of ESG (Primary/Revisional), Technique Used, Type and Class of Associated Medication, Dose (mg/week), Duration of Use, Timing of Medication Initiation (Simultaneous or After ESG), Control Group, and Risk of Bias Assessment, as well as outcome measures such as %TWL (Total Weight Loss), %EWL (Excess Weight Loss), Mean BMI Reduction (kg/m^2^), Weight Regain Rate (%), Follow-up Duration (months), Statistical Significance (p), Fasting Glucose, HbA1c, Lipid Profile (Total Cholesterol, HDL, LDL, Triglycerides), Blood Pressure (SBP/DBP), Inflammatory Markers (CRP, IL-6), Associated Comorbidities (T2DM, Hypertension, Dyslipidemia), and Reduction or Withdrawal of Previous Medications.

### Data synthesis

The meta-analysis was conducted using a random-effects model to account for heterogeneity across studies, pooling mean differences between endoscopic sleeve gastroplasty (ESG) combined with anti-obesity medication and ESG alone. Separate analyses were performed for HbA1c variation (%) and total weight loss (%TWL), based on the mean and standard deviation reported in the included studies. For studies reporting interquartile ranges, values were converted to standard deviations using established statistical methods. The meta-analysis was performed using the R statistical software (version 4.4.1), employing the “meta” and “metafor” packages to calculate pooled estimates, confidence intervals, and heterogeneity metrics (Q, τ^2^, and I^2^) under a random-effects model.

## Results

**Fig. 1:**
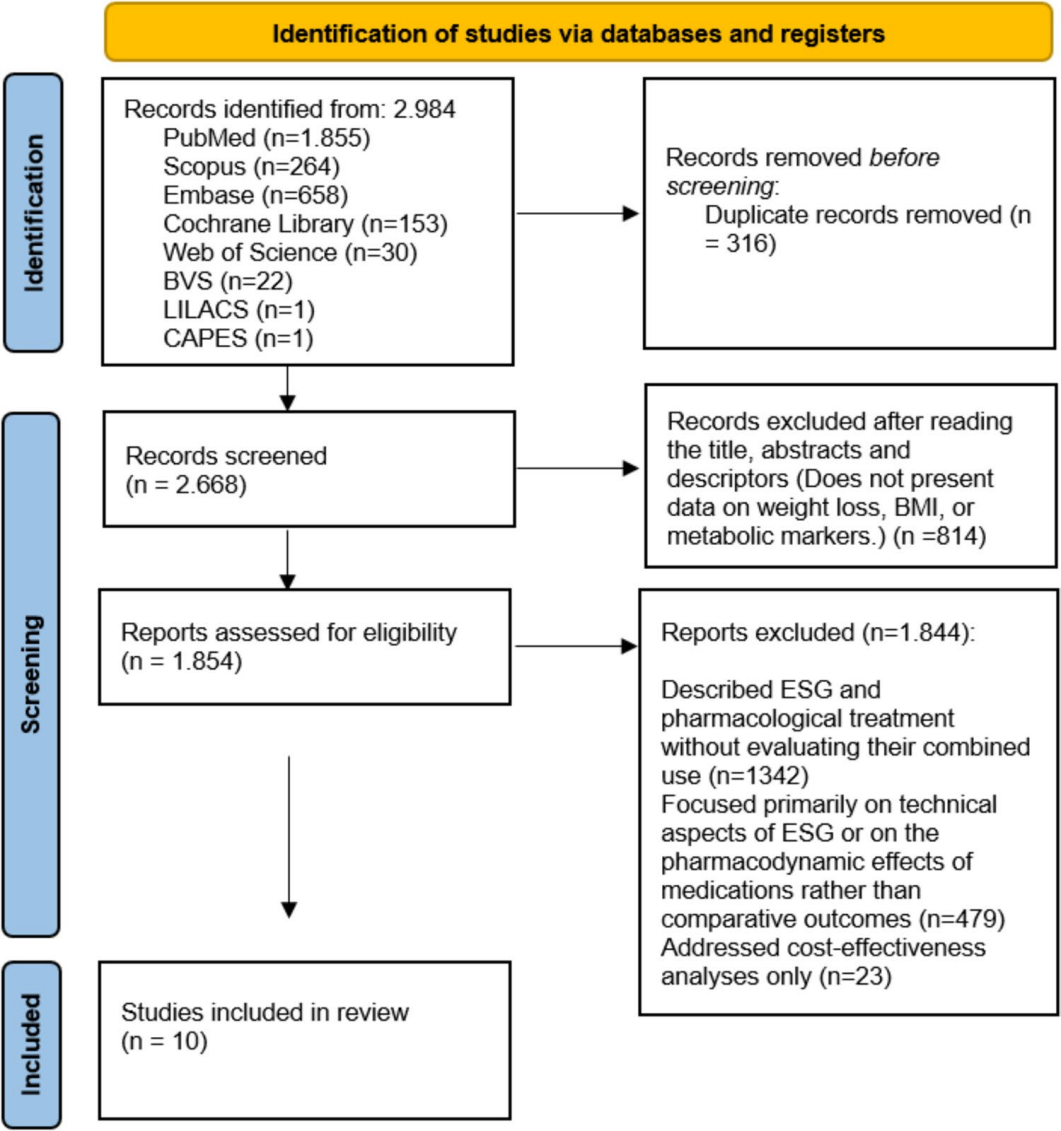
Article eligibility screening process

The complete screening and selection process, illustrated in Fig. 1, identified 2984 records across eight databases and, after excluding 1844 reports that did not meet the eligibility criteria, resulted in the inclusion of 10 studies [13, 14, 22–29] in the final review.

Although publication year was not an eligibility criterion, all included studies were published within the last five years (2020–2025) and collectively encompassed a total sample of 578 patients. The characteristics of the interventions are presented in Table 1.

**Table 1:** Characteristics of the interventions

Author/Year	Population (ESG + Medication)	Type of ESG (Primary/Revisional)	Technique used	Type of associated medication	Pharmacological class	Dose (mg/week)	Duration of use	Start timing of medication (simultaneous or After ESG)	Control group	Risk of bias assessment (NOS)
Chung et al. (2025)	8	Primary ESG — all patients underwent initial (not revisional) endoscopic sleeve gastroplasty for obesity management.	Apollo OverStitch Sx suturing system (Apollo Endosurgery Inc.).	Oral semaglutide (Rybelsus, Novo Nordisk Inc.).	Glucagon-like peptide-1 (GLP-1) receptor agonist.	14 mg/day (equivalent to 98 mg/week).	From the second week after ESG until the 6-month follow-up.	After ESG — medication started two weeks post-procedure.	ESG alone (patients who underwent endoscopic sleeve gastroplasty without anti-obesity medication).	Good
Badurdeenet al. (2020)	*n* = 30 (before matching) /*n* = 26 (after matching)	Primary ESG	Apollo OverStitch (implied by standard ESG technique)	Liraglutide	GLP-1 receptor agonist	Variable doses evaluated (no significant difference between doses reported; specific dose not stated)	7 months after initiation (liraglutide introduced 12 months post-ESG)	After ESG (12 months post-ESG)	ESG alone (matched control)	Good
Gala et al. (2024)	204 patients (13.5% of 1506 total). Type of ESG: Primary (no mention of revisional procedures).	Primary (no mention of revisional procedures).	Apollo OverStitch (standard ESG technique, implied by methodological context).	Anti-obesity medication (AOM)	GLP-1 receptor agonists and Non-GLP-1 AOMs.	–	Up to 24 months of follow-up	Variable — simultaneous or post-ESG, according to categories (None, X < 0 days, X < 6 months, 6 ≤ X < 12 months, X ≥ 12 months).	ESG alone (No AOM) GLP-1 vs. other AOMs. Different start times of AOM therapy.	Good
Lahooti et al. (2025)	22 patients with obesity and MASLD (Metabolic dysfunction-associated steatotic liver disease)	Primary ESG	Full-thickness endoscopic suturing device (Apollo OverStitch).	Semaglutide.	Incretin mimetic / GLP-1 receptor agonist.	–	12 months of follow-up	Simultaneous with ESG (“ESG + GLP1-RA combination”).	ESG alone, Medication (Semaglutide) alone.	Good
Jirapinyo et al. (2023)	69 patients received combination therapy (21 with GLP-1RA; 48 with non–GLP-1RA AOMs).	–	Suturing (24–21%) and plication (76–79%) ESG techniques were used (likely Apollo OverStitch system; inferred from suturing ESG standard).	Semaglutide or liraglutide likely, phentermine/topiramate, topiramate, phentermine, bupropion/naltrexone, bupropion.	GLP-1RA → incretin mimetics Phentermine/topiramate → sympathomimetic + anticonvulsant Topiramate → anticonvulsant Bupropion/naltrexone → dopamine/norepinephrine reuptake inhibitor + opioid antagonist Phentermine → sympathomimetic amine	–	–	Combination Therapy: within 6 months before or after ESG Sequential Therapy: more than 6 months before or after ESG	ESG monotherapy group (no AOMs).	Good
Hoff et al. (2024)	48 patients	Revisional (performed after sleeve gastrectomy due to weight regain).	Endoscopic Sleeve Gastroplasty (ESG) – Apollo OverStitch system (information implied by standard technique)	Liraglutide or Semaglutide.	GLP-1 receptor agonists.	–	Up to 24 months (follow-up data reported up to 24 months).	Within 6 months after R-ESG (not simultaneous).	R-ESG alone (without GLP-1).	Good
Hoon Kim et al. (2024)	62 patients	Primary ESG	Endoscopic sleeve gastroplasty (device not specified, likely Apollo OverStitch based on standard practice at Weill Cornell Medicine).	Semaglutide, phentermine (with or without topiramate), bupropion (with or without naltrexone), orlistat, liraglutide, metformin, and topiramate alone	GLP-1 receptor agonists (semaglutide, liraglutide), sympathomimetic amine (phentermine), anticonvulsant (topiramate), dopamine/norepinephrine reuptake inhibitor (bupropion), opioid antagonist (naltrexone), lipase inhibitor (orlistat), and biguanide (metformin)	–	–	Before and/or after ESG (sequential pharmacotherapy)	ESG alone	Good
Hoon Kim et al. (2024)	83 patients	Primary ESG	Endoscopic sleeve gastroplasty (device not specified, likely Apollo OverStitch based on standard practice at Weill Cornell Medicine).	Semaglutide, phentermine (± topiramate), bupropion (± naltrexone), orlistat, liraglutide, metformin, topiramate alone.	GLP-1 receptor agonists (semaglutide, liraglutide), sympathomimetic amines (phentermine), anticonvulsant (topiramate), antidepressant (bupropion), opioid antagonist (naltrexone), lipase inhibitor (orlistat), biguanide (metformin).	–	–	Sequential — medication initiated after ESG.	ESG alone.	Fair
Hoff et al. (2021)	27 patients	Primary ESG.	Endoscopic Sleeve Gastroplasty with standardized suturing patterns (Apollo OverStitch)	Semaglutide	GLP-1 receptor agonist.	Initial dose 0.25 mg/week, titrated up to a maximum of 1.5 mg/week.	Not directly stated; semaglutide initiated after 5 months post-ESG, continued during the study follow-up	After ESG (post-procedure; started after the 5th month).	Sham group (placebo; 28 participants, mean age 36 years; 21 women).	Good
Lahooti et al. (2025)	25 patients	Primary ESG	-	Semaglutide, Liraglutide, Tirzepatide	GLP-1 receptor agonists	–	–	Two combination groups — GLP-1 initiated before ESG GLP-1 initiated after ESG	ESG alone (*n* = 263)	Good

*ESG* endoscopic sleeve gastroplasty, *R-ESG* revisional endoscopic sleeve gastroplasty, *AOM* anti-obesity medication, *GLP-1* glucagon-like peptide-1, *GLP-1RA* glucagon-like peptide-1 receptor agonist, *Apollo OverStitch Sx* OverStitch Surgical Suturing System, *MASLD* metabolic dysfunction-associated steatotic liver disease, *SG* sleeve gastrectomy, *DNRI* dopamine/norepinephrine reuptake inhibitor, mg/week expressed as milligrams per week: International Units per Week, pharmacotherapy initiated before or after ESG in a non-simultaneous manner: Sequential Therapy, placebo control group used for comparison:Sham Group, *NOS* Newcastle–Ottawa Quality Assessment Scale, metformin class:Biguanide, phentermine class:Sympathomimetic Amine, topiramate class:Anticonvulsant, naltrexone class:Opioid Antagonist, orlistat class:Lipase Inhibitor, pharmacological class that includes GLP-1 receptor agonists:Incretin Mimetics

A total of 10 distinct anti-obesity medications were reported in association with endoscopic sleeve gastroplasty (ESG) across the 10 eligible studies, yielding 32 occurrences overall. The most frequently reported agents were semaglutide (8 occurrences; 25.0%), liraglutide (6; 18.8%), topiramate (5; 15.6%), naltrexone (3; 9.4%), phentermine (2; 6.3%), bupropion (2; 6.3%), orlistat (2; 6.3%), metformin (2; 6.3%), tirzepatide (1; 3.1%), and unspecified anti-obesity medication (1; 3.1%). The primary efficacy outcomes are summarized in Table 2.

**Table 2:** Primary efficacy outcomes

Author/Year	%TWL (total weight loss)	%EWL (excess weight loss)	Mean BMI reduction (kg/m^2^)	Maintenance of weight loss (Time)	Weight regain rate (%)	Follow-up duration (months)	Statistical significance (p)	Overall efficacy comment
Chung et al. (2025)	1 mo: 8.35% (Combo) vs 4.32% (ESG alone); 3 mo: 13.27% vs 8.56%; 6 mo: 20.27% vs 15.32%	1 mo: 31.95% (Combo) vs 23.32% (ESG alone); 3 mo: 50.66% vs 38.45%; 6 mo: 62.66% vs 52.67%	From 33.15 ± 2.16 to 26.43 ± 0.78 (Combo group); Δ = –6.72 kg/m^2^, *p* < 0.001	Maintained through 6 months post-procedure; no mention of loss rebound during follow-up	–	6	TBWL: 1 mo *p* = 0.005, 3 mo *p* = 0.013, 6 mo *p* = 0.015; EBWL: 1 mo *p* = 0.023, 3 mo *p* = 0.024, 6 mo p = 0.034; BMI reduction: *p* < 0.001	The combination of ESG + oral semaglutide (Rybelsus 14 mg/day) significantly enhanced total and excess weight loss and HbA1c reduction compared to ESG alone, without increasing adverse events. Efficacy was superior at all follow-up points (1, 3, and 6 months).
Badurdeenet al. (2020)	2 mo: 18.43 ± 1.55 4 mo: 22.02 ± 1.84 7 mo: 24.72 ± 2.12	4 mo: 75.32 ± 14.19 7 mo: 84.33 ± 14.57	2 mo: 6.61 ± 0.77 4 mo: 7.90 ± 0.95	–	–	7 months post liraglutide start (total 19 months post-ESG)	Yes — multiple comparisons significant (*p* < 0.05 to < 0.001)	The combination of ESG and liraglutide resulted in significantly greater weight loss, BMI reduction, and visceral fat decrease than ESG alone, without additional serious adverse events.
Gala et al. (2024)	GLP-1: -13.7% (6m), -14.9% (12m), -16.3% (18m), -16.7% (24m). Other AOMs: -13.9% (6m), -17.1% (12m), -14.4% (18m), -14.2% (24m).	Significant difference at 24 months (*p* = 0.007).	–	Sustained up to 24 months; best maintenance observed when AOM was initiated after 6 months.	No regain observed in the group that started AOM after 6 months.	24	TBWL: *p* = 0.003 (6m), *p* = 0.014 (24m). EWL: *p* = 0.007 (24m). No significant difference between medication classes (*p* > 0.05).	GLP-1 users showed a trend toward greater weight loss at 18 and 24 months, though not statistically significant. Late initiation of AOM (>6 months after ESG) was associated with better weight maintenance and absence of regain. The proportion of responders (≥10% TBWL) was similar between AOM and no-AOM groups (≈70%, p=1.000).
Lahooti et al. (2025)	ESG alone: 13.3% (IQR 5.11–18.64) Semaglutide alone: 5.33% (IQR 1.76–10.63) ESG + GLP1-RA: 12.45% (IQR 4.9–18.4) p = 0.0037 for ESG vs Semaglutide alone (significant); p = 0.1273 for ESG vs ESG+GLP1-RA (non-significant).	–	ESG alone: –5.39 (IQR –6.69 to –1.95) Semaglutide alone: –1.77 (IQR –3.91 to –0.55) ESG + GLP1-RA: –5.48 (IQR –6.3 to –2.65) p < 0.0001 for ESG vs Semaglutide (significant); *p* = 0.1664 for ESG vs ESG+GLP1-RA (non-significant).	12 months	–	12 months	HSI (ESG vs Semaglutide): *p* = 0.0068 %TBWL (ESG vs Semaglutide): *p* = 0.0037 No significant difference between ESG + GLP1-RA and ESG alone across measured variables.	ESG alone achieved superior weight loss and hepatic steatosis index (HSI) reduction compared to Semaglutide alone, indicating higher global efficacy. The combination ESG + GLP1-RA did not show statistically significant improvement over ESG alone but may benefit lipid profile management.
Jirapinyo et al. (2023)	ESG + GLP-1RA: 23.7 ± 4.6% ESG + non–GLP-1RA: 18% ESG monotherapy: 17.3% Sequential ESG then AOM: 14.7% Sequential AOM then ESG: 12%	–	–	12 months.	–	12 months.	GLP-1RA combination vs. others: *p* < 0.0001 to p = 0.04 (significant superiority of ESG + GLP-1RA) ≥10% TWL: *p* = 0.02	Combination of ESG with GLP-1RA achieved the highest weight loss efficacy and 100% success rate (≥10% TWL) at 12 months. Sequential therapy, particularly AOM preceding ESG, yielded the lowest efficacy (12% TWL). ESG + GLP-1RA combination significantly outperformed ESG monotherapy and other AOM combinations.
Hoff et al. (2024)	6 months: 12.61 ± 2.06% (R-ESG + GLP-1) vs 10.44 ± 2.30% (R-ESG) 12 months: 18.01 ± 2.43% vs 15.92 ± 2.13% 24 months: 18.50% vs 15.77 ± 3.95% (*p* = 0.006)	–	Baseline BMI: 37.65 ± 2.97	Maintained through 24 months.	–	24 months.	%TWL at 24 months = *p* = 0.006 %BFM at 12 months = *p* = 0.017 %BFM at 24 months = *p* = 0.562 TBWL > 10% = *p* = 0.234 TBWL > 15% = *p* = 0.018 TBWL > 20% = *p* < 0.001	The combination of R-ESG with GLP-1 agonists resulted in significantly greater total body weight loss and a higher proportion of patients achieving > 15% and > 20% TBWL at 24 months, suggesting a synergistic effect between endoscopic and pharmacologic therapy for weight regain after sleeve gastrectomy.
Hoon Kim et al. (2024)	–	–	–	–	–	From June 2021 to September 2023 (up to 27 months)	Total visits/year: *p* = 0.01 Nutrition visits/year: *p* = 0.06 (approached significance) Gastroenterology nurse practitioner visits/year: *p* = 0.02 Gastroenterology physician visits/year: *p* = 0.02	Combination therapy (ESG + pharmacotherapy) significantly increased post-procedure visit compliance compared to ESG alone, particularly for gastroenterology MD and NP follow-ups, which may contribute to improved outcomes and better adherence.
Hoon Kim et al. (2024)	–	–	–	–	–	–	No statistically significant difference between ESG alone and ESG + pharmacotherapy for FIB-4 score: Pre-ESG *p* = 0.56 Post-ESG *p* = 0.88 Mean change *p* = 0.49	There was no clinically or statistically significant difference in the FIB-4 score between ESG alone and ESG combined with pharmacotherapy. Both groups showed a slight, non-clinically relevant increase in FIB-4 (<0.1). Authors suggest hepatic improvement may require a longer follow-up period to manifest.
Hoff et al. (2021)	26.7 ± 1.8% in the Semaglutide group vs. 19.6 ± 1.3% in the Sham group (*p* < 0.0001).	86.3 ± 11.8% in the Semaglutide group vs. 60.4 ± 9.4% in the Sham group (*p* < 0.001).	–	–	–	–	*p* < 0.0001 for %TWL; *p* < 0.001 for %EWL; *p* = 0.0394 for HbA1c change.	The combination of ESG with semaglutide enhanced total and excess weight loss, approximating the average results typically observed after laparoscopic sleeve gastrectomy (LSG). It also significantly reduced body fat mass (12.7 ± 2.1% vs. 9 ± 1.8%, p < 0.001) without safety concerns, demonstrating high treatment adherence and improved glycemic control.
Lahooti et al. (2025)	ESG alone: 13.51 ± 8.92% GLP-1 before ESG: 11.61 ± 6.92% GLP-1 after ESG: 11.88 ± 8.27%	–	–	12 months	–	12 months	Mean TBWL differences not significant: *p* = 0.434 (GLP-1 before ESG), *p* = 0.447 (GLP-1 after ESG) Proportion achieving >10% TBWL: *p* = 0.030 (significant, favoring ESG alone)	ESG monotherapy achieved superior results, with 72.2% of patients reaching >10% total body weight loss at 12 months, compared to 53.8% in both combination therapy groups. The sequence of GLP-1 therapy (before or after ESG) did not significantly affect outcomes. Authors note that patients requiring combination therapy might represent a subgroup with more resistant weight-loss trajectories.

Total Weight Loss (*%TWL* Total Weight Loss), Excess Weight Loss *(%EWL* Excess Weight Loss), *BMI* Body Mass Index, kilograma por metro quadrado (*kg/m*^*2*^ kilograms per square meter), Follow-up Duration (months, follow-up in months); Interquartile Range (*IQR* Interquartile Range), Statistical Significance (*p-value* probability value), Glucagon-Like Peptide-1 Receptor Agonist (*GLP-1RA* Glucagon-Like Peptide-1 Receptor Agonist), Anti-Obesity Medications (*AOMs* Anti-Obesity Medications), Total Body Weight Loss *(%TBWL* Total Body Weight Loss), Hepatic Steatosis Index (*HSI* Hepatic Steatosis Index), Body Fat Mass (*%BFM* Percentage of Body Fat Mass), Sleeve Gastrectomy (*SG* Sleeve Gastrectomy, Revisional Endoscopic Sleeve Gastroplasty (*R-ESG* Revisional Endoscopic Sleeve Gastroplasty), Non–Glucagon-Like Peptide-1 Anti-Obesity Medications (*non–GLP-1RA AOMs* Non–GLP-1 Receptor Agonist Anti-Obesity Medications), Nurse Practitioner (*NP* Nurse Practitioner), Gastroenterology Medical Doctor (*MD* Gastroenterology Physician), Fibrosis-4 Index (*FIB-4* Fibrosis-4 Index, Laparoscopic Sleeve Gastrectomy (*LSG* Laparoscopic Sleeve Gastrectomy); glycated hemoglobin (*HbA1c* glycated hemoglobin).

The metabolic and clinical outcomes are presented in Table 3. The eligible studies provided limited and heterogeneous reporting of these variables, with the most consistently documented outcomes being HbA1c (%) and associated comorbidities, particularly type 2 diabetes mellitus (T2DM), hypertension, and dyslipidemia.

**Table 3:** Metabolic and clinical outcomes

Author/Year	Fasting glucose (variation)	HbA1c (%)	Total cholesterol	HDL	LDL	Triglycerides	Blood pressure (SBP/DBP)	Inflammatory markers (CRP, IL-6, etc.)	Associated comorbidities (T2DM, Hypertension, Dyslipidemia)	Reduction/Withdrawal of previous medications
Chung et al. (2025)	–	Mean reduction after 6 months: 1.2 ± 0.13% (ESG alone) vs 1.4 ± 0.19% (ESG + AOM) — *p* = 0.02	–	–	–	–	–	–	Dyslipidemia improvement reported: 40% (ESG alone) vs 50% (ESG + AOM) (*p* = 0.67). Diabetes control improved (HbA1c ↓). Hypertension and other comorbidities not specified.	–
Badurdeenet al. (2020)	–	5.09 ± 0.41 (ESG+L) vs 5.40 ± 0.45 (ESG alone), *p* = 0.013	–	–	–	–	–	–	HTN 53%, OSA 48.5%, Dyslipidemia 48.5%, NAFLD 36.4%, Prediabetes 33.3%, Arthropathy 27.3%, PCOS 18.2%, CAD 3%	–
Gala et al. (2024)	–	–	–	–	–	–	–	–	–	–
Lahooti et al. (2025)	–	ESG alone: Δ –0.3 (IQR –1.3 to 0.1) Semaglutide alone: Δ –0.3 (IQR –1.0 to 0.1) ESG + GLP1-RA: Δ –0.25 (IQR –0.7 to 0.0) *p* > 0.05 (not significant).	–	–	ESG alone: Δ +2.5 (IQR –10.5 to 21.5) Semaglutide alone: Δ –7.7 (IQR –20.8 to 9.0) ESG + GLP1-RA: Δ –4.5 (IQR –27.5 to 23.3) *p* = 0.0108 (significant reduction only in Semaglutide group).	ESG alone: Δ –45.5 (IQR –92.6 to 9.2) Semaglutide alone: Δ –23.6 (IQR –74.5 to 51.5) ESG + GLP1-RA: Δ –38 (IQR –67.5 to 8.8) *p* = 0.18–0.59 (not significant).	–	–	Obesity with MASLD; subset with Type 2 Diabetes (48.1% ESG alone, 25.9% ESG+GLP1-RA, 61.7% Semaglutide alone).	–
Jirapinyo et al. (2023)	-	–	–	–	–	–	–	–	52% had diabetes or prediabetes.	–
Hoff et al. (2024)	–	–	–	–	–	–	–	–	–	–
Hoon Kim et al. (2024)	–	–	–	–	–	–	–	–	–	–
Hoon Kim et al. (2024)	–	–	–	–	–	–	–	–	–	–
Hoff et al. (2021)	–	Decrease of 0.95 ± 0.35% in the Semaglutide group vs. 0.61 ± 0.77% in the Sham group (*p* = 0.0394).	–	–	–	–	–	–	–	–
Lahooti et al. (2025)	–	–	–	–	–	–	–	–	–	–

Fasting Glucose (fasting glucose level); glycated hemoglobin (*HbA1c* glycated hemoglobin A1c), High-Density Lipoprotein (*HDL* high-density lipoprotein cholesterol), Low-Density Lipoprotein (*LDL* low-density lipoprotein cholesterol), Systolic Blood Pressure (*SBP* systolic blood pressure), Diastolic Blood Pressure (*DBP* diastolic blood pressure), C-Reactive Protein (*CRP, C-* reactive protein), Interleukin-6 (*IL-6* interleukin-6), Type 2 Diabetes Mellitus (*T2DM* type 2 diabetes mellitus), Obstructive Sleep Apnea (*OSA* obstructive sleep apnea), Nonalcoholic Fatty Liver Disease (*NAFLD* nonalcoholic fatty liver disease), Metabolic Dysfunction-Associated Steatotic Liver Disease (*MASLD* metabolic dysfunction-associated steatotic liver disease), Polycystic Ovary Syndrome (*PCOS* polycystic ovary syndrome), Coronary Artery Disease (*CAD* coronary artery disease), Interquartile Range (*IQR* interquartile range).

**Fig. 2:**
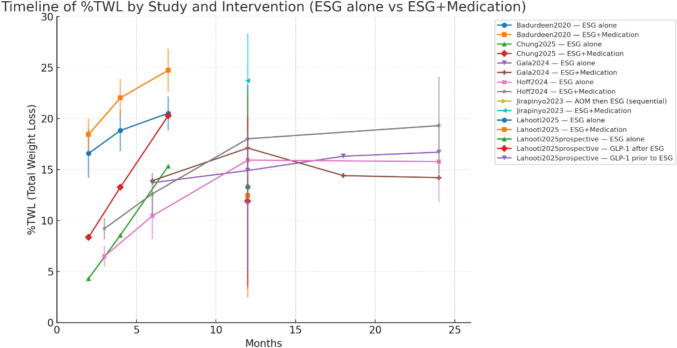
Short-and long-term weight loss outcomes comparing ESG alone versus ESG combined with anti-obesity medication

The Fig. 2 shows a consistent short-term advantage for combining ESG with medication: multiple studies (Chung, 2025, Badurdeen, 2020, Hoff, 2024) report significantly greater %TWL for ESG + medication at early follow-up (roughly 2–7 months) and Hoff2024 shows the difference persists through 12–24 months. However, longer term results are heterogeneous—Gala, 2024 reports the combination outperforming at 12 months but not at 18–24 months, while Lahooti2025 found no significant difference at 12 months and, in a larger cohort, a higher proportion achieving >10% TBWL with ESG monotherapy (suggesting selection bias or that patients placed on combination therapy were harder to treat). Jirapinyo, 2023 shows notably large 12 month losses with combination therapy (23.7% ± 4.6), highlighting variability by center, medication type, and patient selection. The plotted error bars reflect reported or approximated dispersion, but missing sample sizes and heterogeneous endpoints, medication classes (GLP 1 RAs vs other AOMs), and timing (concurrent vs sequential) limit direct pooling or definitive causal claims.

**Fig. 3:**
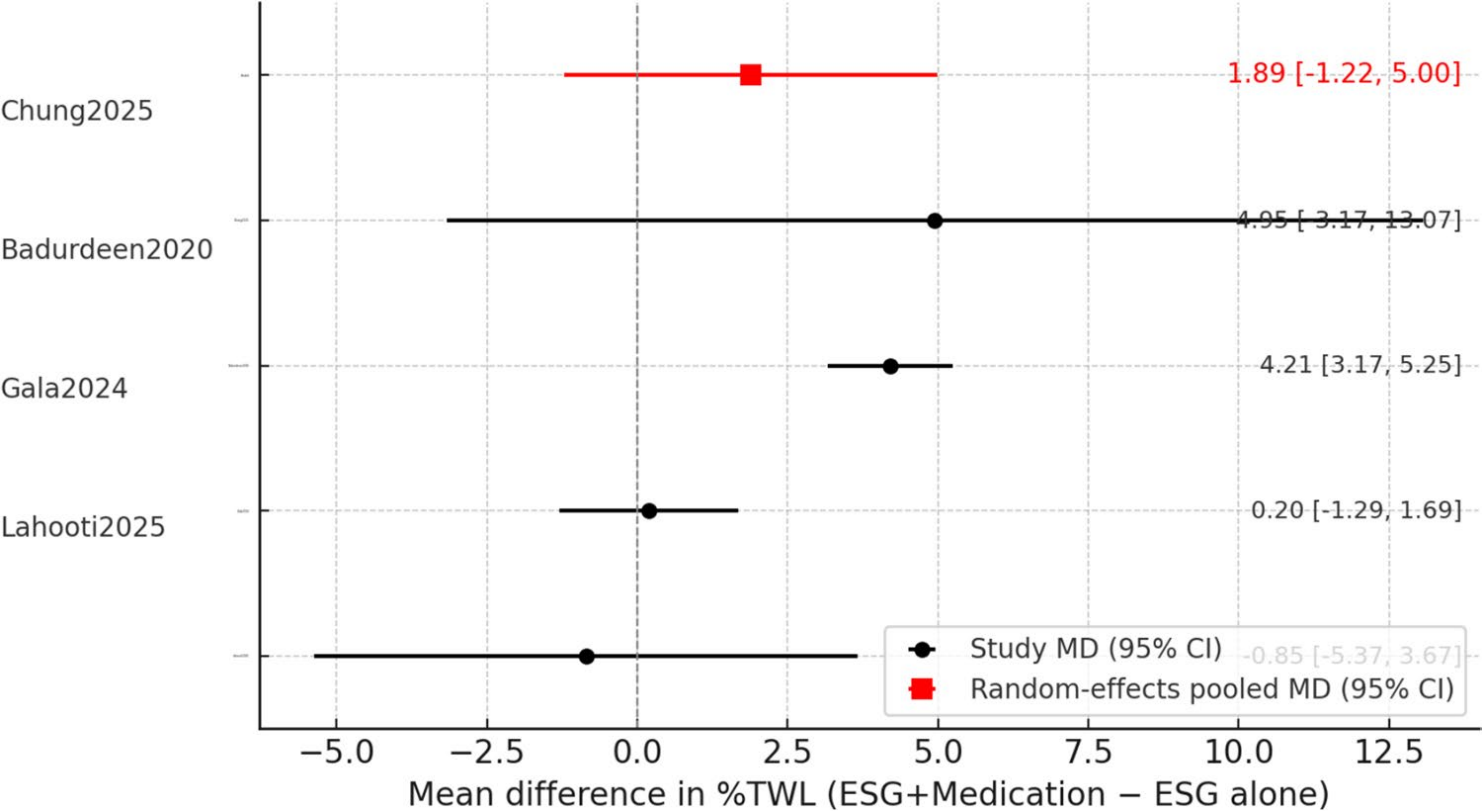
Pooled analysis of %TWL difference between ESG alone and ESG combined with medication

The forest plot (Fig. 3) shows a small pooled advantage for ESG plus medication at ~7 months (mean difference ≈ +1.9% TWL favoring combination), but this effect is not statistically significant (95% CI − 1.22 to 4.99) and is of uncertain clinical importance. Study results are inconsistent: Badurdeen, 2020 and Chung, 2025 show substantial benefits with combination therapy, Lahooti, 2025 slightly favors ESG alone, and Gala, 2024 is essentially neutral. Between study heterogeneity is very high (*I*^2^ approximately 80–90%; tau^2^ ≈ 6.9), reflecting differences in study size, patient selection, medication type/timing, and variability in reported dispersions.

**Fig. 4:**
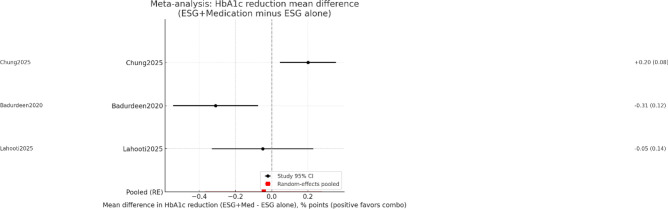
Random-effects meta-analysis of mean HbA1c change: ESG alone versus ESG combined with anti-obesity medication

The Fig. 4 displays the random-effects meta-analysis summarizing the mean difference in HbA1c change between ESG combined with anti-obesity medication and ESG alone, based on three included studies. The pooled mean difference is − 0.04 percentage points (95% CI − 0.37 to 0.28; *p* = 0.79), i.e., essentially no difference in HbA1c reduction between combination therapy and ESG alone. Individual study effects are inconsistent: Chung2025 favors combination (+0.20), Badurdeen2020 favors ESG alone (− 0.31), and Lahooti2025 is nearly neutral (− 0.05). Heterogeneity is high (*Q* = 13.08; *I*^2^ ≈ 85%; τ^2^ ≈ 0.069), indicating substantial between‑study inconsistency. Practically, the pooled estimate is both statistically non‑significant and clinically negligible (differences < 0.5% HbA1c are generally not considered clinically meaningful).

## Discussion

Across the included studies, short-term outcomes consistently demonstrated greater total body weight loss (%TWL) among patients receiving ESG combined with anti-obesity medication compared with ESG alone; however, a more nuanced interpretation of these findings suggests that this early advantage should be understood within the context of temporal dynamics, patient selection, and treatment heterogeneity. In the randomized and observational datasets by Chung et al. [13], combination therapy yielded significantly higher mean %TWL at each follow-up point (8.35 vs 4.32 at 2 months (*p* = 0.005), 13.27 vs 8.56 at 4 months (*p* = 0.01), and 20.27 vs 15.32 at 7 months (*p* = 0.02)) demonstrating an early and sustained advantage for adjunct pharmacotherapy. Similarly, Badurdeen et al. [22] reported that patients treated with ESG + medication achieved markedly greater weight loss compared with ESG alone, with differences of approximately +1.9 to +4 percentage points in TWL across 2-, 4-, and 7-month intervals (all *p* < 0.01). In contrast, larger cohorts such as Gala et al. [14] and Lahooti et al. [28] showed attenuated or non-significant differences at 12–24 months, suggesting not only regression toward parity over time but also the possibility that early benefits may reflect transient pharmacological amplification rather than durable synergistic effect. When pooled in the present meta-analysis, the mean difference favored ESG + medication by approximately +1.9 % TWL (95 % CI − 1.22 to 4.99), a modest, statistically non-significant effect of uncertain clinical importance. Taken together, these data suggest that the addition of pharmacotherapy may potentiate early weight loss after ESG; however, this effect appears highly dependent on follow-up duration, medication characteristics, and patient-related factors, and therefore cannot be assumed to translate into sustained clinical superiority.

Several hypotheses may explain why the apparent early advantage of combination therapy is not consistently maintained over time. First, heterogeneity in the type, potency, and mechanism of action of anti-obesity medications, particularly between GLP-1 receptor agonists and other pharmacological classes, may lead to variable durability of effect across studies [13, 14, 22, 23]. Second, differences in the timing of medication initiation, whether concomitant or delayed after ESG, may influence early versus late weight trajectories, with initial pharmacological intensification potentially diminishing as treatment adherence fluctuates or as dose adjustments occur. Third, variability in patient selection, including baseline disease severity, metabolic profile, and prior treatment history, may introduce confounding, particularly in observational cohorts where combination therapy is more likely to be prescribed to patients perceived as higher risk or more difficult to treat. Fourth, regression of the initial effect may reflect physiological adaptation, behavioral factors, or reduced adherence over time, all of which are well-recognized in chronic weight management. Finally, selection bias inherent to non-randomized designs may contribute to apparent early differences that attenuate with longer follow-up. Collectively, these factors highlight that the observed pattern is unlikely to be explained by a single mechanism, but rather by the interaction of pharmacological, procedural, and patient-level determinants.

Regarding metabolic outcomes, the analysis revealed minimal and inconsistent differences in glycemic improvement between ESG alone and ESG combined with pharmacotherapy, further reinforcing the need for cautious interpretation. In Chung et al. [13], the combination group achieved a slightly greater mean HbA1c reduction (1.4 ± 0.19 %) compared with ESG alone (1.2 ± 0.13 %), a difference that, although statistically significant (*p* = 0.02), remains clinically modest given the small absolute change (< 0.5 %). Conversely, Badurdeen et al. [22] reported a paradoxical finding in which ESG monotherapy showed greater HbA1c reduction (5.40 ± 0.45 %) than the combination group (5.09 ± 0.41 %; *p* = 0.013), suggesting that metabolic response may not parallel weight loss patterns and may be influenced by additional confounders. In Lahooti et al. [28], both approaches produced comparable outcomes (Δ – 0.3 % vs – 0.25 %; *p* > 0.05), confirming the overall neutrality of metabolic effects. The pooled random-effects model demonstrated a non-significant mean difference of – 0.04 percentage points (95 % CI – 0.37 to 0.28; *p* = 0.79), with substantial heterogeneity (I^2^ ≈ 85 %), indicating marked inconsistency across studies. These findings suggest that any early advantage in weight reduction does not reliably translate into superior glycemic outcomes, thereby limiting the justification for combined therapy as a strategy for metabolic optimization.

Although the combination of ESG and AOMs is conceptually appealing, given the complementary mechanisms of gastric restriction and neuro-hormonal modulation [24–29], the present findings reinforce an important methodological principle: biological plausibility does not equate to demonstrated clinical superiority. While it is reasonable to hypothesize that the integration of mechanical and pharmacological interventions could enhance and sustain weight loss [13, 14, 22, 23], the current body of evidence does not consistently support this assumption when subjected to comparative analysis. From a clinical standpoint, this implies that the use of concomitant pharmacotherapy should not be interpreted as inherently superior, but rather as a potentially context-dependent strategy whose benefits may vary according to patient characteristics, treatment timing, and therapeutic goals.

The present study also offers several strengths that contribute to its relevance within the existing literature. First, it provides a focused and structured synthesis of a clinically relevant question that has not been adequately addressed by prior reviews, specifically the direct comparison between ESG + AOM versus ESG alone. Second, it incorporates both published studies and grey literature, allowing a more comprehensive mapping of an emerging field in which evidence remains limited and rapidly evolving. Third, by explicitly demonstrating the heterogeneity, limitations, and uncertainty of the current data, the study contributes not only by identifying potential signals of benefit but also by delineating the boundaries of what can be reliably inferred. In this sense, its contribution is both descriptive and critical, helping to organize fragmented evidence into a coherent analytical framework.

From a practical perspective, the findings of this review should be interpreted as supporting a more cautious and individualized approach to the use of anti-obesity medications in conjunction with ESG. Rather than endorsing routine combination therapy, the available evidence suggests that pharmacological adjuncts may be considered in selected patients, particularly those at higher risk of suboptimal response or weight regain, while acknowledging that the magnitude and durability of benefit remain uncertain. This has direct implications for clinical decision-making, including patient counseling regarding realistic expectations, consideration of treatment burden and cost, and the need to balance potential short-term gains against long-term uncertainty. Importantly, the present analysis does not establish a definitive treatment algorithm but instead informs a decision that is already being made in clinical practice under conditions of limited evidence, thereby contributing to more evidence-aware and context-sensitive care.

The study highlights several priorities for future research. There is a clear need for well-designed, multicenter randomized controlled trials with standardized pharmacotherapy protocols, clearly defined timing of medication initiation, and extended follow-up to assess durability of outcomes. Future investigations should also address key sources of heterogeneity, including differences in medication class, dosing strategies, and patient selection criteria. In addition, emerging areas such as predictive modeling and artificial intelligence may play an important role in identifying patient subgroups most likely to benefit from combination therapy, enabling more precise and personalized treatment strategies. Beyond efficacy, future research should incorporate domains related to quality of care, safety, adherence, and health system impact, thereby providing a more comprehensive evaluation of the role of combined ESG and pharmacotherapy in contemporary obesity management.

## Limitation

Of the ten included studies, three were full-text publications and seven were conference abstracts. This represents a limitation, as the predominance of conference abstracts restricts the depth of methodological appraisal, the completeness of outcome reporting, and the overall confidence that can be placed in the synthesized evidence. Because abstracts often provide condensed data, important aspects such as detailed study design, patient selection procedures, treatment protocols, follow-up consistency, and risk-of-bias domains could not always be assessed with the same level of scrutiny as in full-text publications. In addition, the small number of complete studies, the predominance of observational data, and the marked clinical and statistical heterogeneity across included investigations further limit direct comparability and the strength of causal inference. The lack of uniformity across reported variables, such as study design, follow-up duration, and medication timing, further constrained comparability between studies. Additionally, not all eligible investigations presented adequate quantitative information to support combined statistical analyses, including the meta-analysis. Variability in medication class, timing of pharmacotherapy initiation, and patient profiles may also have contributed to inconsistent estimates across studies and may limit the generalizability of the findings to broader clinical settings. Nevertheless, these limitations should be interpreted within the context of an emerging field in which directly comparative evidence remains scarce. In this sense, the present review is relevant not because it provides definitive answers, but because it organizes fragmented evidence, clarifies the current boundaries of inference, and identifies the methodological priorities required for future research. These methodological and reporting inconsistencies underscore the need for future research with standardized protocols, comprehensive data reporting, and full-text publication to strengthen the reliability of evidence in this emerging field.

## Conclusion

Combination therapy with ESG and anti-obesity medications may enhance early weight loss; however, consistent long-term superiority over ESG alone has not been demonstrated and appears dependent on medication class, timing, and patient characteristics. Clinically, concomitant pharmacotherapy should be considered selectively, rather than as a universally superior strategy. Given the limited and heterogeneous evidence base, further well-designed multicenter randomized trials with standardized protocols and longer follow-up are needed to clarify durability, cost-effectiveness, and patient subgroups most likely to benefit.

## Data Availability

This study’s results are supported by data from earlier published sources. Extracted and analyzed datasets are available from the corresponding author upon reasonable request.
